# Inhibitory and Toxic Effects of Volatiles Emitted by Strains of *Pseudomonas* and *Serratia* on Growth and Survival of Selected Microorganisms, *Caenorhabditis elegans*, and *Drosophila melanogaster*

**DOI:** 10.1155/2014/125704

**Published:** 2014-06-11

**Authors:** Alexandra A. Popova, Olga A. Koksharova, Valentina A. Lipasova, Julia V. Zaitseva, Olga A. Katkova-Zhukotskaya, Svetlana Iu. Eremina, Alexander S. Mironov, Leonid S. Chernin, Inessa A. Khmel

**Affiliations:** ^1^Institute of Molecular Genetics, Russian Academy of Sciences, Kurchatov Square 2, Moscow 123182, Russia; ^2^M.V. Lomonosov Moscow State University, A.N. Belozersky Institute of Physico-Chemical Biology, Leninskie Gory 1-40, Moscow 119991, Russia; ^3^State Research Institute of Genetics and Selection of Industrial Microorganisms, Moscow 117545, Russia; ^4^Engelhardt Institute of Molecular Biology, Russian Academy of Sciences, Vavilov Street 32, Moscow 119991, Russia; ^5^Department of Plant Pathology and Microbiology, The Robert H. Smith Faculty of Agriculture, Food and Environment, the Hebrew University of Jerusalem, 76100 Rehovot, Israel

## Abstract

In previous research, volatile organic compounds (VOCs) emitted by various bacteria into the chemosphere were suggested to play a significant role in the antagonistic interactions between microorganisms occupying the same ecological niche and between bacteria and target eukaryotes. Moreover, a number of volatiles released by bacteria were reported to suppress quorum-sensing cell-to-cell communication in bacteria, and to stimulate plant growth. Here, volatiles produced by *Pseudomonas* and *Serratia* strains isolated mainly from the soil or rhizosphere exhibited bacteriostatic action on phytopathogenic *Agrobacterium tumefaciens* and fungi and demonstrated a killing effect on cyanobacteria, flies (*Drosophila melanogaster*), and nematodes (*Caenorhabditis elegans*). VOCs emitted by the rhizospheric *Pseudomonas chlororaphis* strain 449 and by *Serratia proteamaculans* strain 94 isolated from spoiled meat were identified using gas chromatography-mass spectrometry analysis, and the effects of the main headspace compounds—ketones (2-nonanone, 2-heptanone, 2-undecanone) and dimethyl disulfide—were inhibitory toward the tested microorganisms, nematodes, and flies. The data confirmed the role of bacterial volatiles as important compounds involved in interactions between organisms under natural ecological conditions.

## 1. Introduction

Volatile organic compounds (VOCs) are commonly produced by bacteria and fungi and emitted to the environment. These compounds are characterized by low molecular weight and high vapor pressure and may affect microorganisms and plants [1–3]. Moreover, many VOCs play a significant role in the communication between organisms and act as infochemicals [4, 5]. At present, more than 200 microbial VOCs have been identified, but none can be considered exclusively of microbial origin or definitely emitted by a specific microbial species [6].

*Pseudomonas* and* Serratia* strains have been shown to produce VOCs that inhibit the growth of various microorganisms [7–9]. VOCs produced by rhizobacteria are involved in their interaction with plant-pathogenic microorganisms and host plants and have antimicrobial and plant-growth-modulating activities [2, 7, 10, 11]. Some of the VOCs produced by* Pseudomonas* and* Serratia* strains may act as inhibitors of the quorum-sensing cell-to-cell communication network which regulates the production of antibiotics, pigments, exoenzymes, and toxins [12].

VOCs synthesized by the soil-borne* Pseudomonas fluorescens* strain B-4117 and* Serratia plymuthica* strain IC1270 might be involved in the suppression of crown-gall disease caused by* Agrobacterium*. A volatile alkyl sulfide compound, dimethyl disulfide (DMDS), which is the major headspace volatile produced by* S. plymuthica* strain IC1270, was found to be emitted from stem tissues of tomato plants treated with this bacterium [9]. DMDS suppressed the growth of* Agrobacterium* in plate assays, suggesting the involvement of this VOC in the biocontrol activity of strain IC1270 toward crown-gall disease [9]. These data indicate that some bacterial volatiles may help to promote antagonistic activities in strains associated with plants.

Bacterial VOCs can be considered as important components of the complex interactive mechanisms among bacteria and between bacteria and other organisms, including eukaryotes, in their natural environments. In this study, we investigated the effects of VOCs emitted by* Pseudomonas* and* Serratia* strains of various origins—mainly soilborne and rhizospheric isolates from various geographic regions. The total pool and individual VOCs produced by these bacteria were shown to suppress growth or kill a wide range of organisms (bacteria, fungi,* Drosophila*, and nematodes), including some that are harmful to agricultural plants. The data support the idea that in most natural environments, individual organisms can be combined into ecological communities, forming a complex system of interspecies interactions that may have wide-ranging consequences for medicine, agriculture, and ecology [13].

## 2. Materials and Methods

### 2.1. Organisms, Media, and Growth Conditions

The bacterial strains used in this work are listed in Table 1. The* Pseudomonas* and* Serratia* strains were grown in liquid Luria-Bertani broth (LB) or on solid (1.5% w/v agar) Luria-Bertani agar (LA) [14] at 28°C. The strains of cyanobacteria were grown in liquid or on agarized BG11_N_ medium [15] in the light at 25°C.

Strains of the fungi* Rhizoctonia solani, Helminth*о*sporium sativum,* and* Sclerotinia sclerotiorum* from the Collection of the Institute of Molecular Genetics, Russian Academy of Sciences, were grown on potato dextrose agar (PDA, Difco) at 25°C.

The* Caenorhabditis elegans* N2 (wild-type) strain (Collection of the State Research Institute of Genetics and Selection of Industrial Microorganisms, Moscow) was cultured on nematode growth agar medium (NGM) at 20°C on plates inoculated with* Escherichia coli* strain MG1655 as a food source. Nematode larval development includes four stages-L1, L2, L3, and L4. After L4,* C. elegans* worms pass to the reproductive adult stage [16].

*Drosophila melanogaster* line F flies with the w1118 mutation (Drosophila Stock Center, Bloomington, IN) were maintained at 25°C on a yeast/sugar/raisin/agar medium containing 8 g of agar, 60 g of dried yeast, 40 g of sugar, 36 g of semolina, and 40 g of raisins, with water added to 1 liter final volume.

### 2.2. Detection of Growth Suppression and Killing Activities of Volatiles Emitted by* Pseudomonas* and* Serratia* Strains

#### 2.2.1. Antibacterial Activity

The effect of volatile-producing bacterial strains against* Agrobacterium tumefaciens* strain C58 was tested using a dual-culture assay essentially as described by Dandurishvili et al. [9]. Two-compartment plastic Petri plates (92 × 16 mm) were filled with LA, one of compartments was inoculated with VOC-producing strain, while the another one with the target strain, so that only the volatiles emitted by the producer strain could reach the target bacteria. The examined volatile-producing strain was placed (20 *μ*L of overnight culture, 4–6 × 10^7^ cells) in one LA filled section and distributed by microbiological loop on the surface of the agar, while 50 *μ*L of overnight culture of* A. tumefaciens* strain C58 grown in LB, sampled with saline solution (0.85% NaCl) and diluted to about 10^6^ cells/mL, was placed on LA in the another section of the plate. In this and all similar cases described below the plates were tightly sealed with four layers of parafilm to prevent leakage of volatiles and incubated at 28°C. In control plates, one of the LA compartments was similarly seeded with the target strain, while the another one was left empty. The results were analyzed after 2 days of bacterial growth. When cyanobacteria were used as the target, one compartment of the bipartitioned Petri dish was filled with BG11_N_ agarized medium, on which 10 *μ*L drops of* Synechococcus* sp. strain PCC 7942 pregrown in liquid BG11_N_ medium for 7 days at 25°C were applied (~10^5^ cells in a drop). The another compartment of the Petri dish was filled with LA and inoculated with the volatile-producing bacterial strain. Similar plates, but without the volatile-producing strain, were used as a control. All plates were tightly sealed with parafilm and placed in the light for 7 days at 25°C.

#### 2.2.2. Antifungal Activity

Bicompartmentalized plates filled with LA on one side and PDA on the another one were used. The LA was seeded with a volatile-producing bacterial strain as described above (Section 2.2.1) and incubated at 28°C. After 24 h of incubation, an agar block (~8 mm in diameter) covered with 5-day-old fungal mycelium was excised and placed onto the PDA-filled section. All plates were tightly sealed with parafilm and incubated at 25°C during 4 days. In the control, the plates were filled with media but the bacteria were omitted.

#### 2.2.3. Activity against Nematodes

One section of the bipartitioned plates was filled with NGM and the another one with LA. The section with NGM was inoculated with* E. coli* strain MG1655 cells, used to feed* C. elegans* strain N2 nematodes, and then 10 hermaphroditic worms at the L4 stage were added on the each Petri dish at the start of experiment. The volatile-producing bacteria were inoculated into the section with LA. The plates were tightly sealed with parafilm and incubated at 24°C, and worm growth and development were analyzed for 8 days. A worm was considered dead when it no longer responded to touch and showed no signs of life during further incubation. In the control, the producing bacteria were omitted. The experiments were repeated twice on three plates per repetition. Adult nematodes, eggs, and L1–L4 forms were counted under the Zoom Stereomicroscope, (Olympus SZ61, Olympus Corporation, Japan); in cases the worms multiplied to large amount the plates were divided into sectors and the numbers of worms were summarized.

#### 2.2.4. Activity against* D. melanogaster*

Test tubes (45 mL) containing yeast/sugar/raisin/agar medium and 10 flies (5 males and 5 females, 10 days of age) were placed into a 340 mL glass container filled with 50 mL LA medium along the sides of the container walls to obtain an agar slants on which the tested VOC-producing bacteria were streaked (see Section 2.2.1). The containers were tightly sealed with parafilm and incubated at 25°C. Growth and development of the flies were analyzed on the fifth day of the experiment. Control experiments were designed similarly but the VOC-producing bacteria were omitted. The experiments were repeated three times, with two test tubes each containing 10 flies per repetition.

### 2.3. Effects of Individual VOCs against Target Microorganisms, Nematodes, and* Drosophila*

The tested chemical standards for individual VOCs in liquid form were DMDS (>99% purity), 2-nonanone (>99%), 2-heptanone (>99%), 2-undecanone (99%), and 1-undecene (98%) (all from Sigma-Aldrich Chimie GmbH, Steinheim, Germany). The action of these compounds on microorganisms, nematodes, and* Drosophila* was determined as described in the previous sections, but instead of bacteria producing volatile substances, chemical preparations of individual VOCs were placed in small foil boxes on LA medium. The plates or containers were tightly sealed with parafilm and incubated at the temperatures indicated above. In controls, the VOCs were omitted. All experiments were repeated three to four times, with two to three plates or tubes per experiment.

### 2.4. HCN Assay

Semiquantitative analysis of cyanide production was made with an Aquaquant-14417.0001 Testsystem (Merck). Cultures of the tested strains were grown 48 h with aeration at 28°C in LB containing 2 g/L of NaCl. Each strain was tested for HCN production in two repeats.

### 2.5. Headspace Solid-Phase Microextraction-Gas Chromatography-Mass Spectrometry (HS SPME-GC-MS)

The procedure was performed as described by Dandurishvili et al. [9]. Briefly, the VOCs in the headspace of bacterial cultures grown on an agar slant (~5 × 10^12^ cells per slanted surface) were analyzed using SPME sample enrichment and GC-MS technique. An Agilent 7890A gas chromatograph equipped with a Combi-PAL autosampler (CTC Analytics AG, Zwingen, Switzerland) and coupled to an Agilent 5975C VL MSD mass spectrometer (Agilent Technologies, Santa Clara, CA) was used for the analysis. The ChemStation (Agilent Technologies) software package was used for instrument control and data analysis. VOCs were tentatively identified (>95% match) based on the National Institute of Standards and Technology/Environmental Protection Agency/National Institutes of Health (NIST/EPA/NIH) Mass Spectral Library (Data Version: NIST 05, Software Version 2.0d) using the XCALIBUR v1.3 program (ThermoFinnigan, San Jose, CA) library. Peak areas of individual compounds were calculated as percentage of the total area of the compounds appearing on the chromatogram. Results are listed as peak area (%) of the headspace. DMDS and 1-undecene, the major components in pool of VOCs emitted by strains* S. proteamaculans* 94 and* P. chlororaphis* 449, respectively, were verified using purchased standards (Alfa Aesar, Karlsruhe, Germany), and their retention indices were calculated according to the retention times of n-alkanes (C4–C12) adjacent to them in the gas chromatogram as described previously [9].

### 2.6. Statistical Analysis

Statistical analyses of experiments were carried out using JMP8 software (SAS Institute Inc., Cary, NC, USA). For the on-plate assays, mean and standard errors were calculated using Windows Excel descriptive statistics program. Differences among data were significant at the level of *P* < 0.05.

## 3. Results

### 3.1. Volatiles Produced by* Pseudomonas* and* Serratia* Strains Suppress Growth of Microorganisms, Nematodes, and* Drosophila*

In a dual-culture test, the following organisms (Table 1) were found capable of producing volatiles that suppress completely or partially growth of* A. tumefaciens* strain C58 (Table 2): rhizospheric* P. chlororaphis* strain 30–84 isolated from the rhizosphere of wheat in Kansas, USA, and six others, isolated from various geographical regions in the former USSR, soilborne strains* P. chlororaphis* 205,* P. fluorescens* B-4117, and* S. plymuthica* strain IC1270 isolated from the rhizosphere of grape, as well as* S. proteamaculans* strain 94 isolated from spoiled meat. In accordance with an earlier report [9], the suppressive effect of the volatiles produced by strains IC1270 and B-4117, as well as by the* P. chlororaphis* strain 449 tested in this work, was bacteriostatic, because* A. tumefaciens* C58 resumed its growth when the parafilm was removed or when the strain was transferred to fresh medium. In addition, we used cyanobacterial strains as other targets. The growth of* Synechococcus* sp. PCC 7942 (Table 2) was strongly inhibited by the volatiles emitted by all tested* Pseudomonas* and* Serratia* strains. In the case of cyanobacteria, the observed effect was bactericidal: transfer of strain PCC 7942 to fresh medium without VOCs did not restore its growth. Similarly pronounced growth suppression by VOCs emitted by* Pseudomonas* (strains 449 and B-4117) and* Serratia* (strains IC1270 and 94) was observed for other cyanobacteria-*Anabaena* sp. PCC 7120,* Nostoc* sp. PCC 6310, and* Nostoc* sp. PCC 9305; however, in those experiments, the level of growth suppression was estimated qualitatively rather than quantitatively because these cyanobacteria form long multicellular filaments, making it difficult to count the exact number of cells.

The total pools of volatiles produced by the tested* Pseudomonas and Serratia* strains were also shown to suppress mycelial growth of the phytopathogenic fungi* Rhizoctonia solani, Helminth*о*sporium sativum,* and* Sclerotinia sclerotiorum* (Table 2). This effect was shown to be fungistatic: when the agar blocks with the target fungus were transferred onto fresh medium without volatiles, the fungi resumed normal growth.

Addition of activated charcoal to adsorb the volatiles emitted by* P. chlororaphis* strain 449 into one section of three-partitioned plates fully eliminated their inhibitory effect on the target strains of* A. tumefaciens*, cyanobacteria, and plant-pathogenic fungi (data not shown). A similar effect of charcoal was described by Dandurishvili et al. [9] to prove the antibacterial and antifungal activities of VOCs produced by* P. fluorescens* strain B-4117 and* S. plymuthica* strain IC1270.

To determine whether bacterial volatiles act on nematodes and fruit flies (*D. melanogaster*), we tested four VOC-producing strains of different species:* P. chlororaphis* strain 449,* P. fluorescens* strain B-4117,* S. plymuthica* strain IC1270, and* S. *р*roteamaculans* strain 94. Treatment by the pool of volatiles emitted by each of these strains irreversibly led to the death of all flies the next day. In controls under the same cultivation conditions but without bacteria, all flies remained alive during at least 5 days of observation. Addition of activated charcoal to the bottom of the container with flies and* P. chlororaphis* strain 449 fully eliminated the inhibitory effect of the volatiles (data not shown).

The effect of the volatiles emitted by the same four tested strains was also investigated on development of the nematode* C. elegans*. In the presence of each of these bacterial strains, the motility of the worms and their rate of reproduction were significantly reduced for 24 to 72 h. The action of the volatiles produced by the bacteria led to retardation of* C. elegans* development as compared to a control without bacteria. The strongest effect was exerted by volatiles emitted by strain IC1270: no egg-hatching or juvenile forms were observed, and both the L4 larval stage and the adult nematodes died over a period of 3–8 days (Table 3).

### 3.2. Detection of VOCs Emitted by* P. chlororaphis* Strain 449 and* S. proteamaculans* Strain 94

Production of VOCs by* S. plymuthica* strain IC1270 and* P. fluorescens* strain B-4117 was identified previously [9]. The main headspace compounds emitted by those strains (around 70 to 90% of all headspace VOCs revealed by GC-MS) were the sulfide VOC DMDS and the hydrocarbon 1-undecene, respectively. Other VOCs were detected in much smaller quantities. Here we investigated the chemical profiles of the VOCs emitted by strains* P. chlororaphis* strain 449 and* S. proteamaculans* strain 94 by headspace-SPME chromatography analysis coupled with software separation of overlapping GC-separated components (Table 4, Supplemented data Figures  S1-A and S1-B, available online at http://dx.doi.org/10.1155/2014/125704). Totally, 14 and 6 compounds, respectively, were identified by GC/MS analysis of VOCs emitted by strains 449 and 94 using the XCALIBUR v1.3 program library. The main VOCs emitted by the* P. chlororaphis* strain 449 were 1-undecene, 2-nonanone, and 2-undecanone. DMDS and 2-heptanone were also produced, but in very low amounts (Table 4, Supplemented data Figure  S1-A). Other compounds were produced in amounts of ~0.1 to 1.4% of the total VOC pool. The composition of VOCs produced by the* S. proteamaculans* 94 strain differed significantly from that emitted by* P. chlororaphis* strain 449. The main headspace VOC emitted by the former was DMDS (Table 4, Supplemented data Figure  S1-B), suggesting it to be the predominant emitted volatile, at least by the tested strains of* Serratia*.

### 3.3. HCN Synthesis of* Pseudomonas* and* Serratia* Strains

Among the volatile substances inhibiting the growth of microorganisms the inorganic volatile compound hydrogen cyanide (HCN) might also have toxic effects on various organisms, including bacteria and plants [17, 18]. Therefore, we tested our VOCs producing strains for ability to produce HCN using strains 30–84 [19] and* P. fluorescens* Pf-5 [20] as positive control, while strain* P. fluorescens* 2–79 [21] as negative control. The results presented in Table 5 demonstrate that* P. chlororaphis* strains 449, 62, 64, 66, and 464 synthesize essential amounts of HCN while two other strains of* Pseudomonas chlororaphis* (445 and 205), as well as* S. proteamaculans* 94 and biocontrol strains of* P. fluorescens* B-4117 and* S. plymuthica* IC1270, almost do not produce it, suggesting that inability to produce HCN does not influence the observed inhibitory effects of volatiles emitted by the tested HCN-negative strains.

### 3.4. Effects of Individual VOCs on Various Test Organisms

The growth inhibition effect of the main individual VOCs (marked in bold in Table 4) was investigated using* A. tumefaciens* strain C58, cyanobacterium* Synechococcus* sp. strain PCC 7942, and the fungus* R. solani* as target microorganisms. The bacteriostatic effect of DMDS on* A. tumefaciens* strain C58, demonstrated previously on several strains of* Agrobacterium* [9], was confirmed in this work. DMDS at 100 *μ*mol completely suppressed the growth of the cyanobacterium strain* Synechococcus* sp. PCC 7942 (Table 6). Significant growth inhibition of strains* A. tumefaciens* strain C58 and* Synechococcus* sp. PCC 7942 and* R. solani* was observed under the action of the ketone 2-nonanone. Another ketone, 2-undecanone (100 *μ*M), completely inhibited the growth of strain* Synechococcus* sp. PCC 7942 and* R. solani*, but did not appreciably affect* A. tumefaciens* strain C58. Although the studied bacteria did not produce 2-heptanone in large quantities, we compared its effect with those of the two other ketones: 2-heptanone had a strong growth-suppressive effect on strains* A. tumefaciens* C58 and* Synechococcus* sp. PCC7942, whereas its effect on* R. solani* was less pronounced. In all cases, the effect of these VOCs toward* R. solani* was fungistatic. Similar fungistatic activity was observed for DMDS toward several plant-pathogenic fungi, including* R. solani* (Dandurishvili and Chernin, unpublished results). 1-Undecene did not significantly affect the growth of any of the three microorganisms tested (Table 6).

Aside from strong antibacterial and antifungal activities, the VOCs studied here had a strong effect on the viability and development of the nematode* C. elegans.* DMDS and the ketones 2-nonanone and 2-undecanone, all at 25 *μ*mol, killed nematodes after 3 days of exposure. In the case of 25 *μ*mol 2-heptanone, 100% of the L4 forms introduced in the experiment turned into adult nematodes during the first 3 days of incubation, but no eggs or juvenile forms appeared. Further incubation killed all of the nematodes. 1-Undecene (25 *μ*mol) inhibited nematode development: on day 3 of incubation, 30% of adult nematodes, 15% of eggs, and no juvenile L1–L3 forms were detected. On day 8, there were 23% adult nematodes, 5% eggs, and 10% juvenile L1–L3 forms; L4 forms were absent. 1-Undecene at 100 *μ*mol killed all nematodes within 3 days.

The strongest effect on* D. melanogaster* viability was manifested by DMDS, 2-heptanone, and 2-nonanone. These VOCs were already killing flies at an amount of 5 to 10 *μ*mol, and 1-undecene killed* Drosophila* at 25–100 *μ*mol. 1-Undecanone had the weakest effect on* Drosophila* (Table 7).

## 4. Discussion

In recent years, the synthesis of VOCs with antimicrobial activity by soil and rhizosphere bacteria has been gaining attention. VOC synthesis has been hypothesized to be a factor in the interactions between bacteria and in their competition with other microorganisms, along with the synthesis of antibiotics, siderophores, and the like [7, 10, 11, 22]. Several bacterial volatiles may have an influence on eukaryotic organisms, including plants and animals, for example,* Arabidopsis thaliana* and* C. elegans* [10]. The actions of individual VOCs of bacterial origin on a wide range of microorganisms have been analyzed in several studies [7–9, 23, 24].

Here, we studied the influence of bacterial VOCs produced by* Pseudomonas* and* Serratia* strains on* A. tumefaciens*, cyanobacteria, fungi,* C. elegans,* and* D. melanogaster*. All of the VOC-producing strains (except* S*.* proteamaculans*) had been previously suggested as potential biocontrol agents of several phytopathogenic bacteria and fungi [9, 25–27], suggesting that the volatiles emitted by these* Pseudomonas* and* Serratia* strains contribute to their biocontrol effect against these plant pathogens. Despite that strain* Serratia proteamaculans* 94 was isolated not from soil/plant habitats we decided to include it in this research because some other strains of this species were isolated from rhizosphere, for example, of oilseed rape [28]. Therefore, strain 94 in our study served as a model to demonstrate that VOCs emitted by this species are able to suppress growth of wide range of microorganisms and even some eukaryotes, including worms and insects. In this work, we showed that the volatiles produced by all tested strains of* Pseudomonas* and* Serratia* inhibit the growth of various fungi,* A. tumefaciens* strain C58, and* Synechococcus* sp. strain PCC 7942.

The inhibitory action of volatiles of the tested strains of* Pseudomonas* and* Serratia* seems to be a cooperative effect of a combination of volatiles produced by the bacteria. We were interested in elucidating the synthesis and action of these bacteria's VOCs. LC-MS/MS analysis revealed VOCs produced by* P. chlororaphis* strain 449 and* S. proteamaculans* strain 94, whereas those emitted by* S. plymuthica* strain IC1270 and* P. fluorescens* strain B-4117 had been detected previously [9]. A study of the action of individual VOCs showed that these compounds participate in growth suppression of the tested organisms.

*S. proteamaculans* strain 94, similar to the previously studied* S. plymuthica* strain IC1270 [9], synthesizes DMDS as the major headspace VOC. This compound was also synthesized by* P. chlororaphis* strain 449, albeit in very small quantities. In contrast to the tested* Serratia* strains,* P. chlororaphis* strain 449 produced several types of ketones. All of these strains had inhibitory effects on bacteria, fungi, flies, and nematodes in dual-culture assays. However, the observed differences between the tested VOC producers in their antagonistic action toward various target organisms may reflect differences in the profile of the emitted active volatile compounds.

Several individual VOCs produced by the studied bacteria demonstrated inhibitory effect on the growth and survival of microorganisms, nematodes, and* Drosophila*. In the case of ketones, the strongest effect on bacteria was demonstrated by 2-nonanone and 2-heptanone. All three ketones exhibited bactericidal activity toward the cyanobacterium* Synechococcus.* It has been recently shown that some VOCs, such as 8-methyl-2-nonanone, 2-decanone, and 3-methyl-1-butanol, display lytic anticyanobacterial activity [29]. Contrary to that, 1-undecene did not suppress the growth of* A. tumefaciens* ([9] and this work), and this work, or the growth of* Synechococcus* or* R. solani*. However, to our surprise, it had a strong killing effect on* D. melanogaster* (Table 7). It also inhibited the development of the nematode* C. elegans.*

*C. elegans* is an attractive model organism to study host-pathogen interactions: it has simple growth requirements, a short generation time, a well-defined developmental process with invariant cell-lineage sorting, a fully sequenced genome, and a suite of well-established genetic tools [30]. Using* C. elegans* as a model, scientists in the last few years have identified a variety of physical, chemical, and biochemical features involved in microbial pathogenesis [31].* C. elegans* is not considered to be a parasite [32], but some aspects of its biology are similar to those of some parasitic nematode groups. The information obtained for* C. elegans* can thus be extrapolated, with caution, to parasitic nematodes [33, 34]. Therefore, we used* C. elegans* as a model organism in our research. Previously, it was shown that some VOCs, including ketones and alcohols, can act as natural chemoattractants or repellents of* C. elegans*, for example, 2-nonanone [35–37]. The present study revealed the killing activity of several VOCs on nematodes.

Strains* P. chlororaphis* 449 and* S. proteamaculans* 94 emitted, respectively, at least 14 and 6 identified compounds that formed peaks in LC-MS/MS analysis (Table 4, Figure S1-A and -B). Obviously, this is only a small proportion of the emitted volatiles detected to date for various bacteria [7, 8, 10, 38], indicating that many other compounds remain to be studied. Of course, we cannot exclude yet that besides that volatiles are tested in this work as individual chemical substances, some other volatiles can contribute to the observed effects being an integrative part of the pool of biologically active volatiles produced by the tested bacteria. One of such volatiles could be hydrogen cyanide (HCN) known as a volatile antibiotic and biocontrol factor of many beneficial rhizosphere strains of* Pseudomonas* species [17, 18, 39]. The results presented in Table 5 demonstrate that five of the tested strains of* P. chlororaphis* synthesized essential amounts of HCN, while two other* P. chlororaphis* strains, as well as strains* P. fluorescens* B-4117*, S. plymuthica* IC1270, and* S. proteamaculans* 94 produce at least not more than traces of this compound. However, entire pools of volatiles emitted by all these strains, regardless of whether or not they produce HCN, exhibited strong inhibitory action on* A. tumefaciens* C58,* Synechococcus, R. solani,* and* H. sativum* (Table 2). Similarly, the HCN-negative strains killed* Drosophila*, indicating that other volatile compounds (e.g., DMDS and some ketones) are responsible for the observed effects.

Production of volatile sulfur compound DMDS is currently under investigation as an alternative to soil fumigation with methyl bromide. DMDS has also been suggested to play a natural defensive role in plant protection, acting as a fumigant [23]. Under the trade name PALADIN, testing of DMDS as a novel preplanting soil fumigant has recently begun. The activity of DMDS in the control of plant-pathogenic fungi [10], weeds [40], and nematodes [41] has been demonstrated. These observations were supported and extended by demonstrating that DMDS can suppress the growth of* Agrobacterium* strains* in vitro* ([9] and this work), as well as mycelial growth of several plant-pathogenic fungi, worms (*C. elegans*), and insects (*D. melanogaster*). Apart from DMDS, other VOCs produced by rhizospheric bacteria, including commercially available volatile antimicrobial compounds, can provide fungistatic and bacteriostatic effects in soil [38]. Inorganic and organic volatile compounds may occur in soil atmospheres in a range of concentrations, and their participation in soil fungistasis has been demonstrated [22]. Different forms of soil sterilization that kill various plant-pathogenic soil inhabitants, such as fungi, bacteria, and nematodes, are a widespread phenomenon [42], presumably mediated by soil microorganisms, including VOC producers. The results presented here extend these observations and indicate the potential of several groups of VOCs emitted by rhizospheric and other microorganisms for the protection of plants, including economically essential crops, against microbial plant pathogens and pathogenic nematodes.

Microbial VOCs have been shown to be able to interact with insects and “insect chemoreception of microbial volatiles may contribute to the formation of neutral, beneficial, or even harmful symbioses and provide considerable insight into the evolution of insect behavioral responses to volatile compounds” [43]. Thus, some VOCs emitted by fungi, for example, 2-octanone, 2,5-dimethylfuran, and 3-octanol, kill* D. melanogaster*, due in part to the generation of reactive oxygen species [44, 45]. However, much less is known about the killing action of VOCs produced by live bacteria on this and other flies. Here we demonstrated the killing effect of volatiles emitted by the tested strains of* Pseudomonas* and* Serratia* on* D. melanogaster*, used as a model insect. The killing activity of several VOCs of bacterial origin against* Drosophila* suggested an additional potential role for VOCs, as protectors of plants against insects [46]. However, to confirm this potential, the insecticide activity of VOCs must be tested against a wide range of plant-attacking bugs. Unfortunately, most of the reports on the biological activity of VOCs are still mainly descriptive. Further studies are required to reveal the chemical processes underlying the observed effects of microbial volatiles on a wide range of target organisms in the natural environment.

## 5. Conclusions

We showed that volatile organic compounds (VOCs) produced by strains of* Pseudomonas* and* Serratia* isolated mainly from rhizosphere of plants are broad range inhibitors of growth of various microorganisms, including plant pathogenic bacteria and fungi and cyanobacteria. LC-MS/MS analysis revealed dimethyl disulfide (DMDS), ketones, and 1-undecene as main headspace VOCs emitted by the tested bacterial strains. A study of the action of individual VOCs showed that these compounds participate in growth suppression of the tested organisms. The results demonstrate that bacterial volatiles are essential components of the chemosphere, which are involved in microbial interactions, particularly in the rhizosphere environment. The observed killing activity of* Pseudomonas* and* Serratia* tested strains as well as DMDS and several ketones against nematodes (*Caenorhabditis elegans*) and flies (*Drosophila melanogaster*) suggested an additional potential of these strains and compounds as protectors of plants against agricultural pests.

## Supplementary Material

Figure S1-A GC/MS chromatogram of VOCs emitted by *P. chlororaphis* 449 (growth on LA 24 h)
Figure S1-B GC/MS chromatogram of VOCs emitted by *S. proteamaculans* 94 (growth on LA 24 h)
S1-A and S1-B, Headspace peak areas of volatiles produced by strains *P. chlororaphis* 449 and *S. proteamaculans* 94, respectively, grown on LA for 24 h at 28°C. VOCs were analyzed by Headspace Solid-Phase Microextraction–Gas Chromatography–Mass Spectrometry (HS SPME–GC–MS) as described in Materials and Methods (p. 2.5) and notes to Table 4. List of identified compounds corresponding to the indicated retention time are presented in Table 4 and shown here above corresponding GC/MS chromatograms for convenience of the reader.

## Figures and Tables

**Table 1:** Bacterial strains used in this work.

Strains	Relevant characteristics	Source or reference
*Pseudomonas *		
*P. chlororaphis* 30–84	Isolated from the rhizosphere of wheat, Kansas, USA	L. Thomashow, USDA-ARS, Pullman, WA, USA
*P. chlororaphis* 449	Isolated from the rhizosphere of maize, Kiev region, Ukraine	[25]
*P. chlororaphis *62	Isolated from the rhizosphere of cotton, Tashkent region, Uzbekistan	[47]
*P. chlororaphis *64	Isolated from the rhizosphere of plantain, Moscow region, Russia	[47]
*P. chlororaphis *66	Isolated from the rhizosphere of alfalfa, Tashkent region, Uzbekistan	[47]
*P. chlororaphis *445	Isolated from the rhizosphere of maize in the Kiev region, Ukraine	[47]
*P. chlororaphis *464	Isolated from the rhizosphere of beet in the Kiev region, Ukraine	[47]
*P. chlororaphis *205	Isolated from soil of rice growing in Kazakhstan	[47]
*P. fluorescens* B-4117	Isolated from soil collected in the Batumi Botanical Garden, Georgia	[9, 26]

*Serratia *		
*S. *р*roteamaculans* 94	Isolated from spoiled meat	[48]
*S. plymuthica* IC1270	Isolated from rhizosphere of grape, Samarkand region, Uzbekistan	[27]

*Cyanobacteria *		
*Synechococcus *sp. PCC 7942	Photoautotrophic cyanobacterium	O.A. Koksharova, Moscow State University, Russia
*Nostoc* sp. PCC 6310	Photoautotrophic and diazotrophic cyanobacterium	U. Rasmussen, Stockholm State University, Sweden
*Nostoc* sp. PCC 9305	Photoautotrophic and diazotrophic cyanobacterium	U. Rasmussen, Stockholm State University, Sweden
*Anabaena *sp. PCC 7120	Photoautotrophic and diazotrophic cyanobacterium	C.P. Wolk, PLR, Michigan, USA

*Other bacteria *		
*Agrobacterium tumefaciens *C58	Nopaline type, isolated from cherry crown gall	[49]
*E. coli *MG1655	F-lambda-*ilvG-rfb-50 rph-1 *	Collection of the Institute of Molecular Genetics RAS
*P. fluorescens *Pf-5	Isolated from rhizosphere of cotton, USA	J. Loper, Oregon State University, Corvallis, OR, USA
*P. fluorescens *2–79	Isolated from rhizosphere of wheat, USA	L. Thomashow, USDA-ARS, Pullman, WA, USA

**Table 2:** Suppression of *Agrobacterium tumefaciens* C58, *Synechococcus* sp. PCC 7942, and fungal growth by volatiles emitted by *Pseudomonas* and *Serratia *strains. The experiments were conducted on three to four plates in each variant and repeated at least twice; total numbers of Petri plates used in each variant are shown in parentheses.

Treatment by volatiles emitted by strains	Treated microorganisms				
	*A. tumefaciens* C58 (CFU)	*Synechococcus* sp. PCC 7942 (CFU)	*R*. *solani* ^a^ (mm)	*S*. *sclerotiorum* ^a^ (mm)	*H*. *sativum* ^a^ (mm)
Control (no treatment)	1.6 ± 0.6 × 10^11^ (9)	4 ± 1 × 10^7^ (9)	14 ± 3 (8)	16 ± 3 (8)	18 ± 3 (8)
*P. chlororaphis* 449	ng (12)	ng (8)	ng (9)	10 ± 2 (12)	6 ± 2 (6)
*P. chlororaphis* 30–84	ng (8)	ng (8)	ng (8)	12 ± 3 (6)	7 ± 2 (9)
*P. chlororaphis* 62	ng (8)	ng (8)	ng (6)	9 ± 2 (8)	4 ± 1 (9)
*P. chlororaphis* 64	ng (6)	ng (8)	ng (6)	10 ± 3 (6)	8 ± 2 (8)
*P. chlororaphis* 66	ng (8)	ng (9)	ng (6)	11 ± 4 (6)	6 ± 2 (9)
*P. chlororaphis* 445	ng (6)	ng (8)	ng (6)	9 ± 2 (6)	3 ± 1 (8)
*P. chlororaphis* 464	ng (6)	ng (8)	ng (6)	9 ± 3 (6)	6 ± 2 (9)
*P. chlororaphis* 205	ng (9)	ng (9)	ng (6)	11 ± 2 (6)	3 ± 1 (9)
*S. proteamaculans* 94	4.5 ± 0.5 × 10^9^ (9)	ng (8)	3 ± 1 (8)	13 ± 3 (8)	5 ± 1 (9)
*P. fluorescens* B-4117	ng (9)	ng (8)	ng (8)	8 ± 2 (12)	4 ± 1 (9)
*S. plymuthica* IC1270	2.5 ± 0.6 × 10^9^ (8)	ng (8)	3 ± 1 (8)	12 ± 2 (8)	9 ± 2 (6)

ng: no visible growth. In controls, plates were filled with corresponding media, but volatile-emitting strains were omitted.

^a^Growth of mycelium measured as distance in mm between the block of fungus and the border of its mycelium.

**Table 3:** Action of volatiles emitted by *Pseudomonas* and *Serratia* strains on *Caenorhabditis elegans*. The numbers of L4 and adult worms, eggs, and L1–L3 forms were counted on the days 3 and 8 after 10 worms of L4 were placed on each culture plate.

Treatment by volatiles emitted by strains	Development of nematodes							
	3 days				8 days			
	L4 forms	Adult nematodes	Eggs	Juvenile L1-L2 forms	Adult nematodes	Eggs	Juvenile L1–L3 forms	L4 forms
*P. chlororaphis* 449	6 ± 2	4 ± 1	1.2 ± 0.2 × 10^2^	14 ± 3 (only L1)	1.3 ± 0.3 × 10^2^	25 ± 5	1.4 ± 0.3 × 10^2^	0
*P. fluorescens *B-4117	0	10	1.5 ± 0.4 × 10^2^	25 ± 5	2 ± 0.5 × 10^2^	~3 × 10^3^	3 ± 1 × 10^2^	1.5 ± 0.5 × 10^2^
*S. plymuthica* IC1270	10	0	0	0	0	0	0	0
*S. proteamaculans* 94	6 ± 2	5 ± 2	14 ± 4	7 ± 3 (only L1)	2 ± 0.4 × 10^2^	1.5 ± 0.4 × 10^2^	1.3 ± 0.3 × 10^3^	0
Control (no treatment)	0	10	3 ± 1 × 10^2^	2 ± 0.6 × 10^2^	4 ± 1 × 10^2^	~4 × 10^4^	~3 × 10^3^	~4 × 10^3^

**Table 4:** Headspace volatiles (Peak Area, %) emitted from bacterial antagonists. Results of three independent experiments with two repetitions for each variant are presented.

Compound*	RT (min)	Strain	
		*P. chlororaphis* 449 (14)**	*S. proteamaculans* 94 (6)
Butanol-1	11.16	1.4***	nd
Methyl thiolacetate	11.80	≤0.1	nd
Isopentanol	12.65	nd	2.2
**Dimethyl disulfide**	12.96	≤0.1	68.7 ± 15.3
**2-Heptanone**	15.73	≤0.1	1.5 ± 0.2
1,5-Dimethylpyrazine	16.21	nd	1.5
**1-Undecene**	18.49	64.5 ± 9.1	nd
**2-Nonanone**	19.28	14.4 ± 5.0	nd
**2-Undecanone**	22.59	12.0 ± 3.6	nd
S-Methyl thiooctanoate	22.68	nd	1.1

*Probability set at >90% to the NIST library, substances marked in bold were additionally tested in this study for biological activity (growth or survival suppression); **total number of identified VOCs produced by the bacterium (see supplement data, Figure S1-A, B); ***mean or mean ± standard error of the Peak Area, % at *P* < 0.05; nd: not detected.

**Table 5:** The production of CN^−^ (mean, *n* = 2) by *Pseudomonas* and *Serratia* strains. *P.  fluorescens* Pf-5 [20] was used as positive while strain *P.  fluorescens* 2–79 as negative controls [21]. HCN production by each strain was detected in two repeats.

Strains	Production of CN^−^, mg/L
*P. chlororaphis *30–84	0.010
*P. chlororaphis* 449	0.020
*P. chlororaphis* 62	0.020
*P. chlororaphis* 64	0.012
*P. chlororaphis* 66	0.035
*P. chlororaphis* 445	0.002
*P. chlororaphis* 464	0.030
*P. chlororaphis* 205	≤0.002
*S. proteamaculans *94	≤0.002
*P. fluorescens *Pf-5	0.030
*P. fluorescens *2–79	0.000
*P. fluorescens *B-4117	≤0.002
*S. plymuthica *IC1270	0.000

**Table 6:** The action of VOCs on *Agrobacterium tumefaciens* C58, *Synechococcus* sp. PCC 7942, and *Rhizoctonia solani*. All experiments were repeated three to four times, with two to three plates per variant. Total number of repetitions for each variant is indicated in parentheses.

VOC	*A. tumefaciens* C58 (CFU)		*Synechococcus* sp. PCC 7942 (CFU)	*R. solani* (mm)	
	Amount of VOC (*μ*mol)				
	10	100	100	10	100
2-Nonanone	2 ± 0.4 × 10^10^ (9)	ng (9)	ng (8)	4 ± 0.9 (6)	ng (6)
2-Heptanone	3 ± 0.2 × 10^9^ (6)	ng (6)	ng (9)	9 ± 4 (6)	4 ± 0.7^a^ (6)
2-Undecanone	4 ± 1 × 10^11^ (9)	3 ± 1 × 10^11^ (9)	ng (8)	6 ± 1.5 (6)	ng (6)
DMDS	4 ± 0.8 × 10^11^ (8)	4 ± 2 × 10^10^ (8)	ng (6)	13 ± 3 (6)	9 ± 3 (6)
1-Undecene	3 ± 0.6 × 10^11^ (8)	3 ± 1 × 10^11^ (8)	2 ± 0.3 × 10^7^ (6)	12 ± 4 (6)	11 ± 2 (6)

ng: no visible growth.

^a^The distance between the block of *R. solani* and the border of its mycelium (mm).

**Table 7:** The action of individual VOCs on *Drosophila melanogaster*. The numbers of live flies per tube of 10 (mean ± SE ) were counted on the 5th day (3 experiments, each with 2 replicate tubes). All flies were alive in control tubes.

VOC	The number of surviving *Drosophila* flies			
	Amount of VOC (*μ*mol)			
	5	10	25	100
DMDS	3 ± 1	0	0	0
2-Nonanone	5 ± 2	3 ± 1	0	0
2-Heptanone	3 ± 1	0	0	0
1-Undecene	10 ± 0	10 ± 0	0	0
2-Undecanone	10 ± 0	9 ± 1	7 ± 2	4 ± 2
