# Supplementary material for: Inhibitory and Toxic Effects of Volatiles Emitted by Strains of Pseudomonas and Serratia on Growth and Survival of Selected Microorganisms, Caenorhabditis elegans, and Drosophila melanogaster
Source: Biomed Res Int. 2014 Jun 11;2014:125704. doi: 10.1155/2014/125704 (PMC4071779; doi:10.1155/2014/125704)
Supplement: Supplementary file 1 — Figure S1-A GC/MS chromatogram of VOCs emitted by P. chlororaphis 449 (growth on LA 24 h) Figure S1-B GC/MS chromatogram of VOCs emitted by S. proteamaculans 94 (growth on LA 24 h) S1-A and S1-B, Headspace peak areas of volatiles produced by strains P. chlororaphis 449 and S. proteamaculans 94, respectively, grown on LA for 24 h at 28°C. VOCs were analyzed by Headspace Solid-Phase Microextraction–Gas Chromatography–Mass Spectrometry (HS SPME–GC–MS) as described in Materials and Methods (p. 2.5) and notes to Table 4. List of identified compounds corresponding to the indicated retention time are presented in Table 4 and shown here above corresponding GC/MS chromatograms for convenience of the reader. [file 125704.f1.pdf]

Supplemental Materials

Fig. S1-A GC-MS chromatogram of VOCs emitted by *P. chlororaphis* 449 (growth on LA 24 h)

**Retention time, min**	**Compound**
11.160	Butanol-1
11.8	Methyl thiolacetate
12.957	**Dimethyldisulfide**
15.732	**2-Heptanone**
17.114	4,4-Dimethyl-2-neopentyl-1-pentene or similar
17.233	2,2,4,6,6-Pentamethylheptene-3 or similar
17.313	(3E)-2,2,3,5,6-Pentamethyl-3-heptene or similar
17.810	2,2,4,6,6-Pentamethyl-3-heptene or similar
17.934	2,2,4,6,6-Pentamethyl-3-heptene or similar
18.340	2,4,4,6,6,8,8-Heptamethyl-1-nonene or similar
18.490	**Undecene-1**
19.284	**2-Nonanone**
22.593	**2-Undecanone**
26.366	(?)-Decen-1-ol acetate or similar

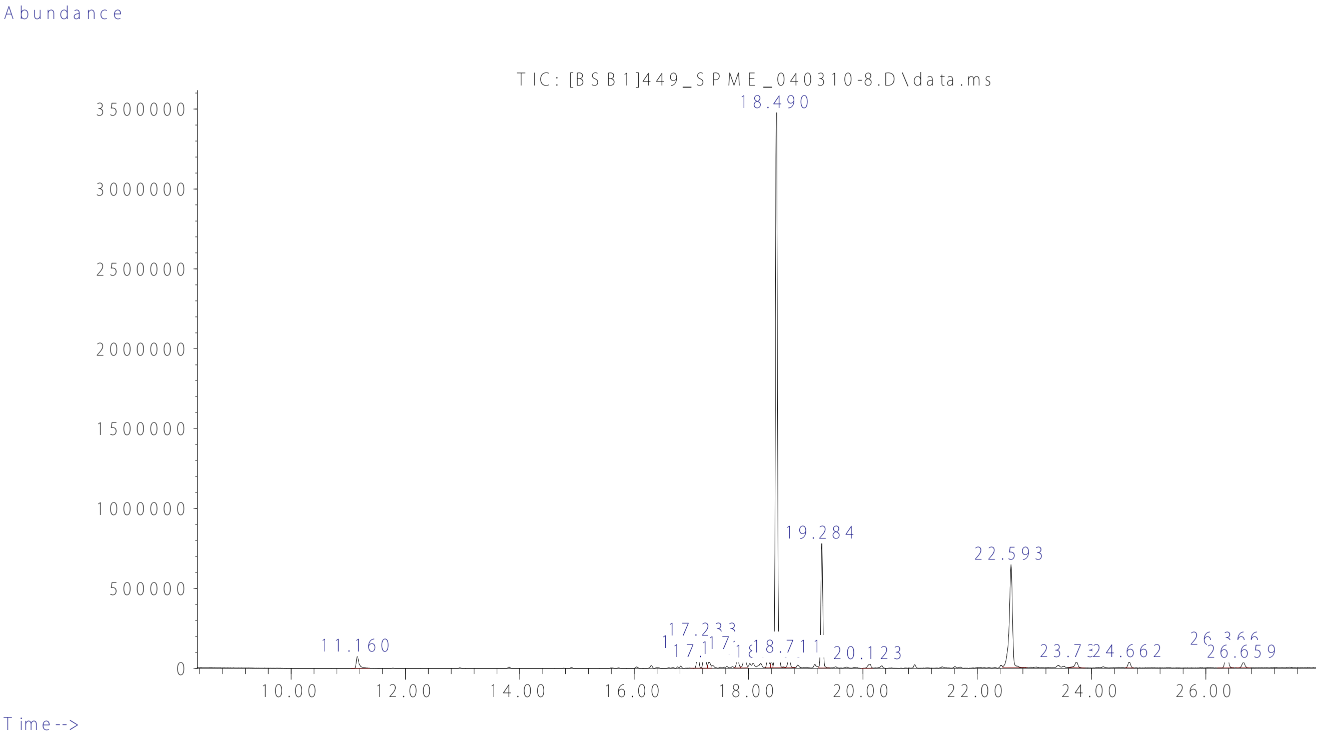

Fig. S1-B GC-MS chromatogram of VOCs emitted by *S. proteamaculans* 94 (growth on LA 24 h)

Retention time, min	Compound
12.653	Isopentanol
12.951	**DMDS**
15.712	**2-Heptanone**
16.215	1,5-Dimethylpyrazine
16.7-22.58	Aliphatic hydrocarbons
22.685	S-Methyl thiooctanoate

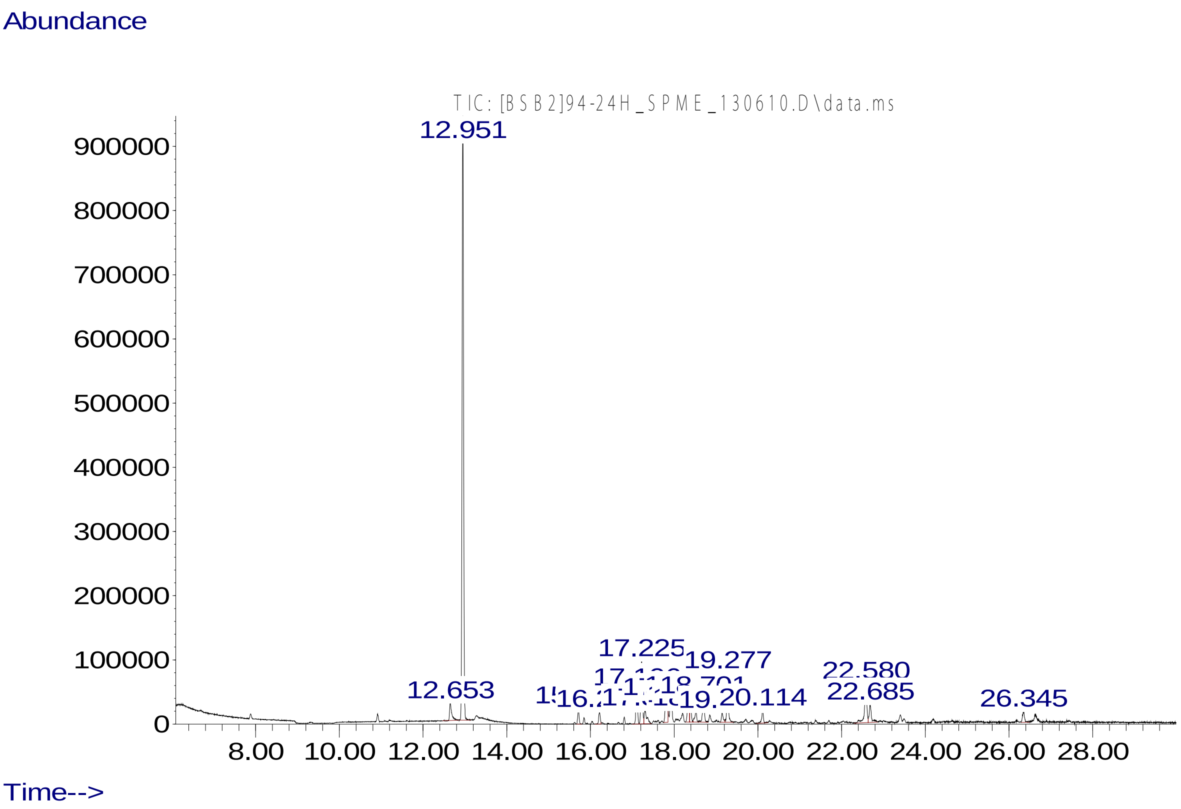

S1-A and S1-B,

Headspace peak areas of volatiles produced by strains *P. chlororaphis* 449 and *S. proteamaculans* 94, respectively, grown on LA for 24 h at 28°C. VOCs were analyzed byHeadspace Solid-Phase Microextraction–Gas Chromatography–Mass Spectrometry (HS SPME–GC–MS) as described in Materials and Methods (p. 2.5) and notes to Table 4. List of identified compounds corresponding to the indicated retention time are presented in Table 4 and shown here above corresponding GC/MS chromatograms for convenience of the reader.
